# Supplementary material for: Predicting unknown binding sites for transition-metal-based compounds in proteins
Source: PLoS One. 2026 Jun 9;21(6):e0349622. doi: 10.1371/journal.pone.0349622 (PMC13249218; doi:10.1371/journal.pone.0349622)
Supplement: S1 Table — For each case, the values reported are: TP/FN (recall). For Metal3D, the thresholds for p(low), p(medium), and p(high) are 0.04, 0.09, and 0.14 for HEWLC, and 0.06, 0.11, and 0.16 for RNaseA. (PDF) [file pone.0349622.s001.pdf]

Table S1: Summary of the true positive (TP), false negative (FN), and recall values for the different predictors and probability thresholds/maps. For each case, the values reported are: TP/FN (recall). For Metal3D, the thresholds for p(low), p(medium), and p(high) are 0.04, 0.09, and 0.14 for HEWLC, and 0.06, 0.11, and 0.16 for RNaseA.

		Metal3D			Metal1D		
Protein	Site	p(high)	p(medium)	p(low)	Zn map	Pt map	Ru map
HEWLC	His15	4/2 (0.66)	6/0 (1.0)	6/0 (1.0)	5/1 (0.83)	5/1 (0.83)	0/6 (0.0)
	Asp101	0/6 (0.0)	0/6 (0.0)	3/3 (0.50)	0/6 (0.0)	0/6 (0.0)	0/6 (0.0)
	Asp119	0/6 (0.0)	0/6 (0.0)	3/3 (0.50)	0/6 (0.0)	0/6 (0.0)	0/6 (0.0)
RNaseA	His119	4/0 (1.0)	4/0 (1.0)	4/0 (1.0)	3/1 (0.75)	0/4 (0.0)	0/4 (0.0)
	His105	0/4 (0.0)	1/3 (0.25)	1/3 (0.25)	0/4 (0.0)	0/4 (0.0)	0/4 (0.0)
	Asp14	0/4 (0.0)	0/4 (0.0)	1/3 (0.25)	0/4 (0.0)	1/3 (0.25)	0/4 (0.0)
