# Supplementary material for: Predicting unknown binding sites for transition-metal-based compounds in proteins
Source: PLoS One. 2026 Jun 9;21(6):e0349622. doi: 10.1371/journal.pone.0349622 (PMC13249218; doi:10.1371/journal.pone.0349622)
Supplement: S2 Table — All structures have been aligned to the backbone of the apo structure (PDB ID 194L). The RMSD corresponding to an occupied binding site is indicated in bold, and an asterisk indicates a rotamer change with respect to the reference (apo) structure. (PDF) [file pone.0349622.s002.pdf]

Table S2: RMSD values for the different X-ray structures considered for the HEWLC. All structures have been aligned to the backbone of the apo structure (PDB ID 194L). The RMSD corresponding to an occupied binding site is indicated in bold, and an asterisk indicates a rotamer change with respect to the reference (apo) structure.

PDB ID	Resolution (Å)	RMSD (Å)				
		backbone	metal binding amino acids	binding sites		
				His15	Asp101	Asp119
194L	1.40	ref.	ref.	ref.	ref.	ref.
2I6Z	1.90	0.25	0.38	**1.14***	0.42	0.22
5II3	1.78	0.25	0.53	**1.18***	1.62*	0.37
5V4G	1.20	0.28	0.54	**1.19***	**1.68***	0.12
6QEA	1.96	0.21	0.54	**1.15***	1.69*	0.25
6WGO	1.30	0.31	0.91	0.24	3.83*	**0.45**
