# Supplementary material for: Predicting unknown binding sites for transition-metal-based compounds in proteins
Source: PLoS One. 2026 Jun 9;21(6):e0349622. doi: 10.1371/journal.pone.0349622 (PMC13249218; doi:10.1371/journal.pone.0349622)
Supplement: S3 Table — All structures have been aligned to the backbone of the apo structure (PDB ID 19PH). The RMSD corresponding to an occupied binding site is indicated in bold, and an asterisk indicates a rotamer change with respect to the reference (apo) structure. (PDF) [file pone.0349622.s003.pdf]

Table S3: RMSD values for the different X-ray structures considered for the RNaseA enzyme. All structures have been aligned to the backbone of the apo structure (PDB ID 19PH). The RMSD corresponding to an occupied binding site is indicated in bold, and an asterisk indicates a rotamer change with respect to the reference (apo) structure.

PDB ID	Resolution (Å)	RMSD (Å)				
		backbone	metal binding amino acids	binding sites		
				Asp14/Met29	His105	His119
1RPH	2.20	ref.	ref.	ref.	ref.	ref.
4S0Q	2.09	0.51	0.68	0.79/**0.91***	0.38	0.44
4S18	2.27	0.48	0.54	**0.72***/**1.1***	**0.29**	**0.33**
5JLG	1.79	0.52	0.48	0.18/0.40	**0.38**	3.09*
