# Supplementary material for: Predicting unknown binding sites for transition-metal-based compounds in proteins
Source: PLoS One. 2026 Jun 9;21(6):e0349622. doi: 10.1371/journal.pone.0349622 (PMC13249218; doi:10.1371/journal.pone.0349622)
Supplement: S4 Table — All structures have been aligned to the backbone of the apo structure (PDB ID 1RPH) and the RMSD is computed for all the atoms of His105 using the structure of PDB 4S18 as reference, for which Metal3D made a successful prediction. (PDF) [file pone.0349622.s004.pdf]

Table S4: RMSD values for the His105 residue in the different X-ray structures considered for the RNaseA enzyme. All structures have been aligned to the backbone of the apo structure (PDB ID 1RPH) and the RMSD is computed for all the atoms of His105 using the structure of PDB 4S18 as reference, for which Metal3D made a successful prediction.

| PDB ID | RMSD (Å) – His105 |
|---|---|
| 1RPH | 0.29 |
| 4S0Q | 0.15 |
| 4S18 | ref. |
| 5JLG | 0.16 |
