# Supplementary material for: Predicting unknown binding sites for transition-metal-based compounds in proteins
Source: PLoS One. 2026 Jun 9;21(6):e0349622. doi: 10.1371/journal.pone.0349622 (PMC13249218; doi:10.1371/journal.pone.0349622)
Supplement: S5 Table — For each case, the values reported are: TP/FN (recall). (PDF) [file pone.0349622.s005.pdf]

Table S5: Summary of the true positive (TP), false negative (FN), and recall values for the sites identified with the cavity-detector predictor Fpocket. For each case, the values reported are: TP/FN (recall).

Protein	Site	Fpocket
HEWLC	His15	3/3 (0.50)
	Asp101	5/1 (0.83)
	Asp119	1/5 (0.17)
RNaseA	His119	1/3 (0.25)
	His105	0/4 (0.0)
	Asp14	0/4 (0.0)
