# Supplementary figures and images for: Predicting unknown binding sites for transition-metal-based compounds in proteins
Source: PLoS One. 2026 Jun 9;21(6):e0349622. doi: 10.1371/journal.pone.0349622 (PMC13249218; doi:10.1371/journal.pone.0349622)

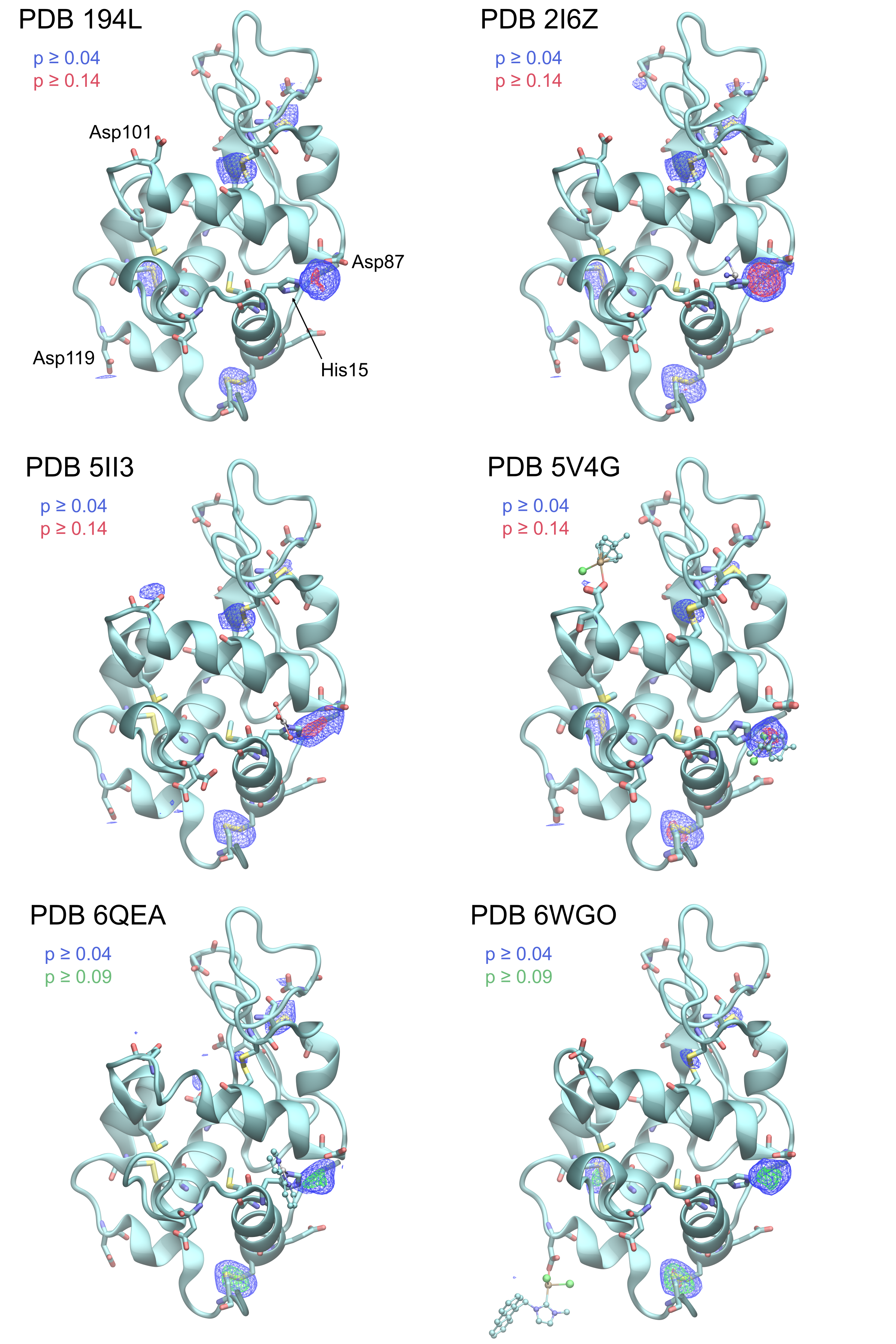

Supplement: S1 Fig — Metal3D probability densities are represented in different colors as isosurfaces with a wireframe representation. The isovalue for the low probability predictions (blue) is 0.04, while the one for higher probability predictions (red) is 0.14. In the cases where no region had probability higher than 0.14, a lower isovalue is used (0.09, green). (PNG) [file pone.0349622.s006.png]

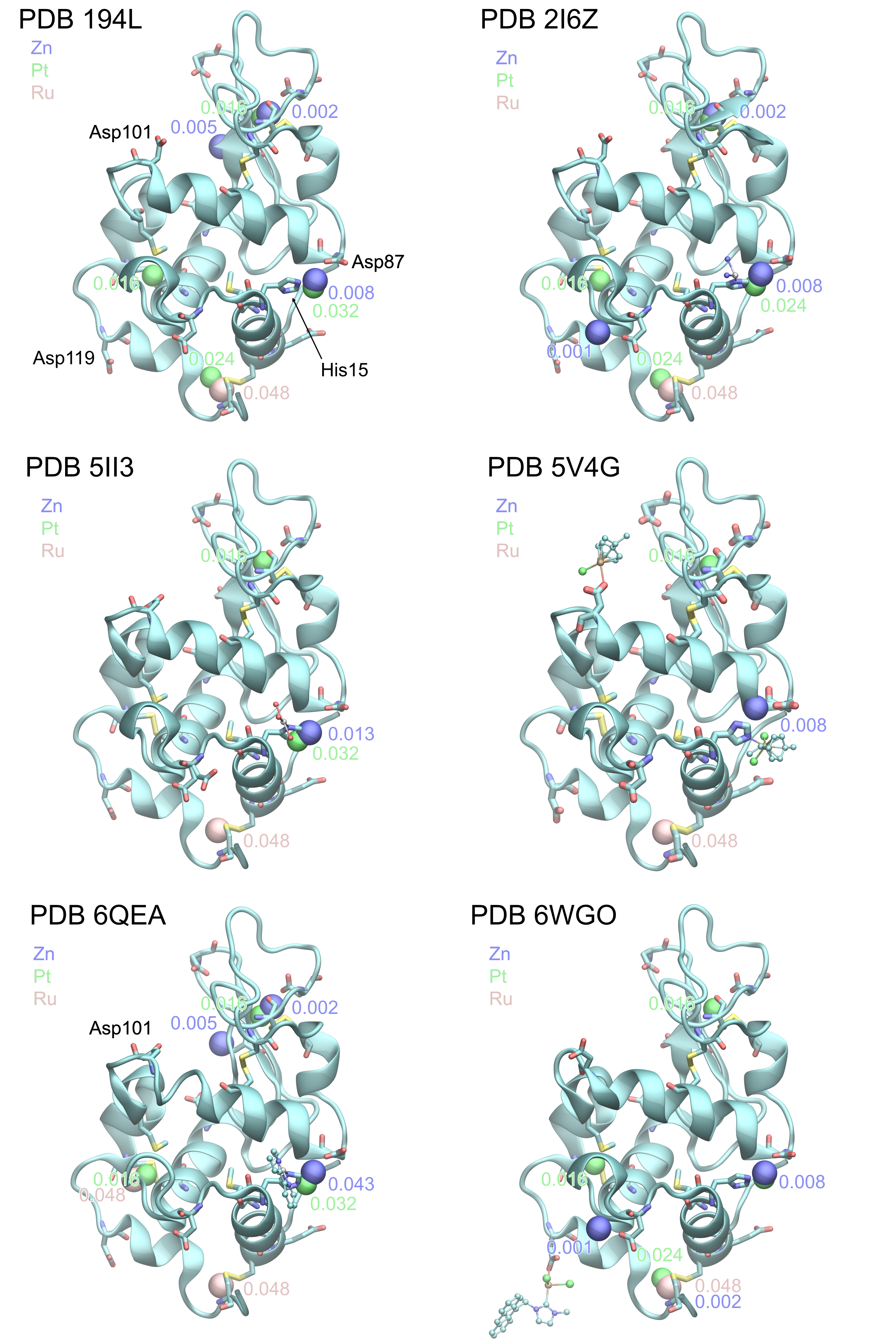

Supplement: S2 Fig — Zinc (blue), platinum (green), and ruthenium (pink). For each prediction, the probability associated with each site is indicated with the same color as the corresponding probability map. (PNG) [file pone.0349622.s007.png]

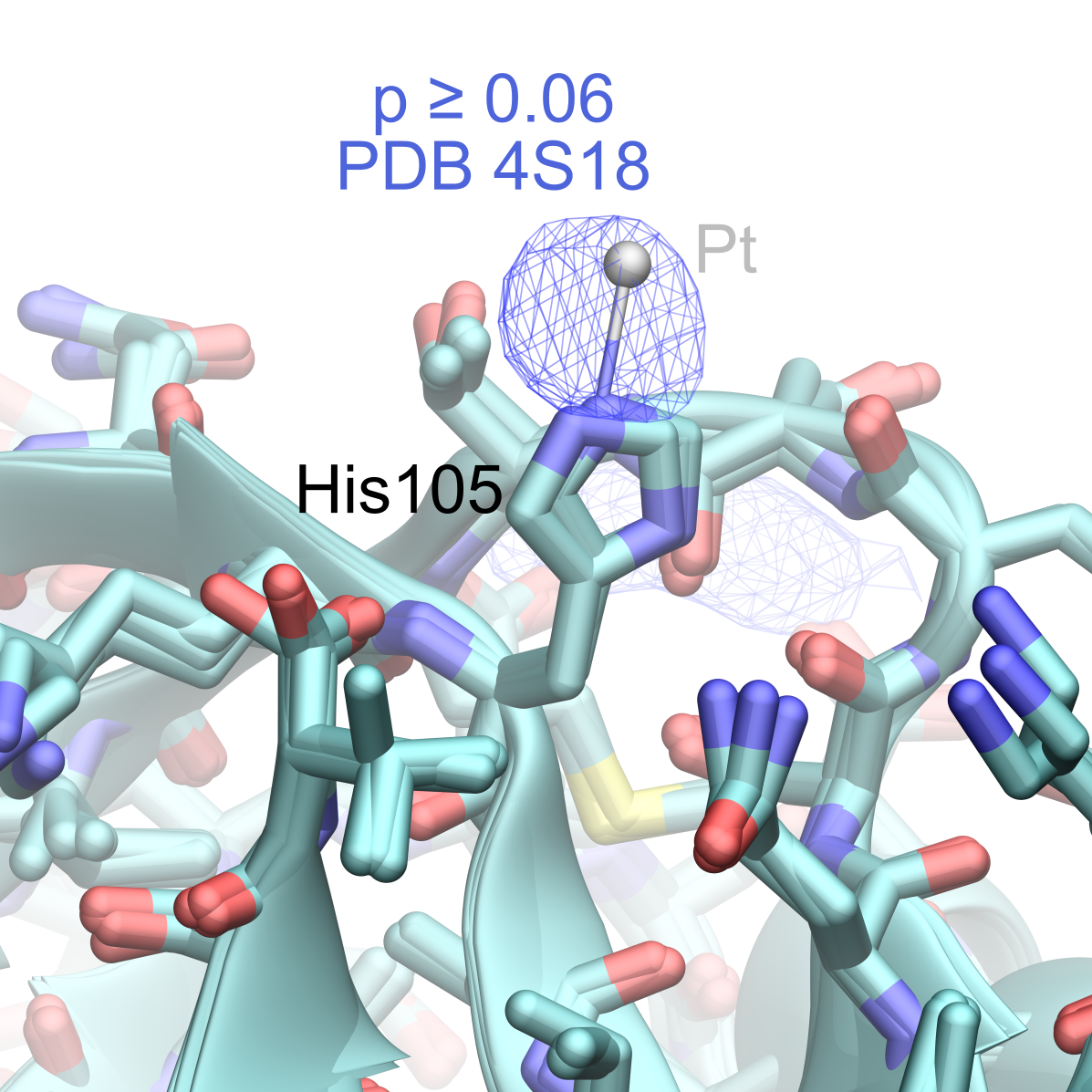

Supplement: S3 Fig — All structures have been aligned to the backbone of the apo structure (PDB ID 19PH), and the probability density generated from Metal3D for PDB 4S18 is also represented in blue. (PNG) [file pone.0349622.s008.png]

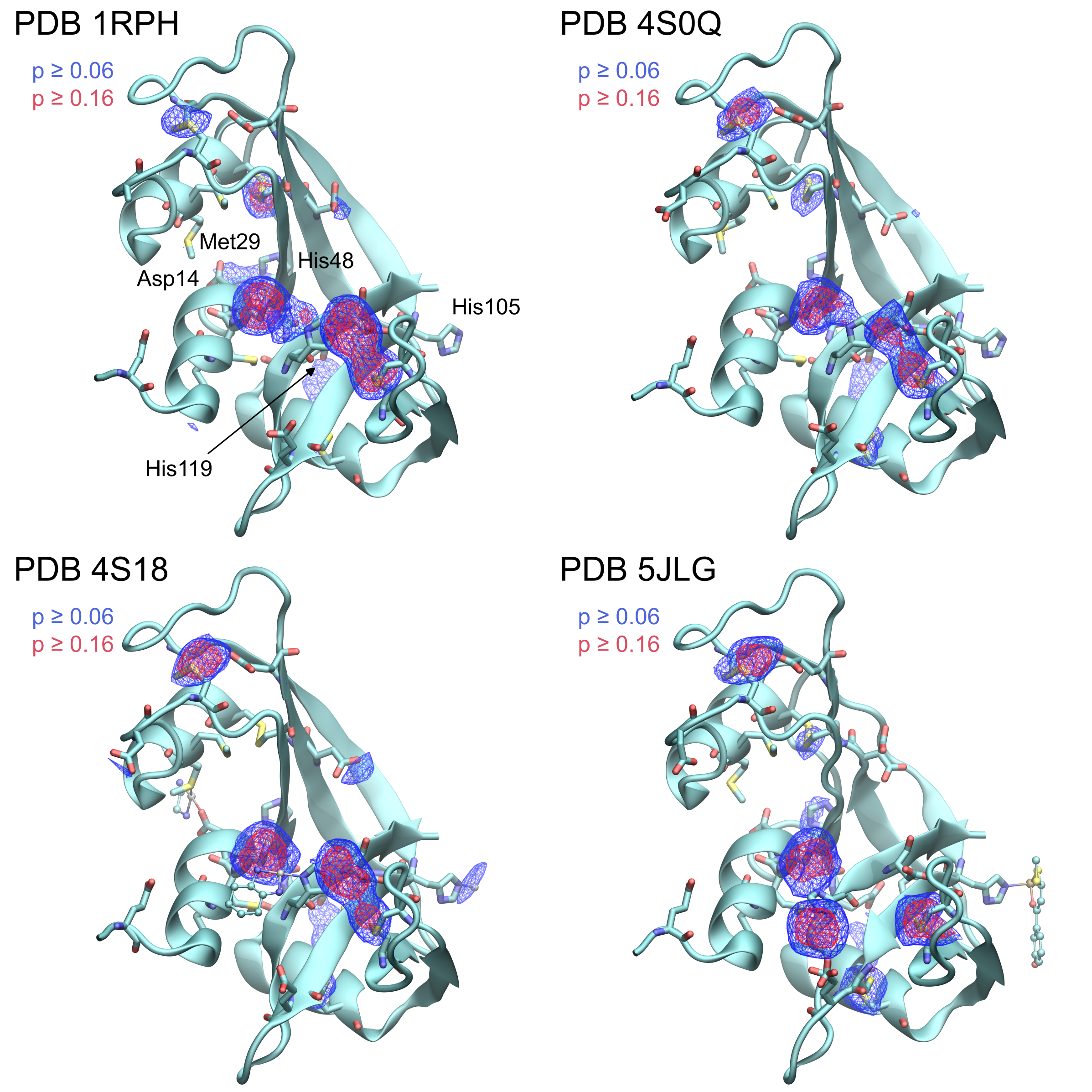

Supplement: S4 Fig — Metal3D probability densities are represented in different colors as isosurfaces with a wireframe representation. The isovalue for the low probability predictions (blue) is 0.06, while the one for higher probability predictions (red) is 0.16. (PNG) [file pone.0349622.s009.png]

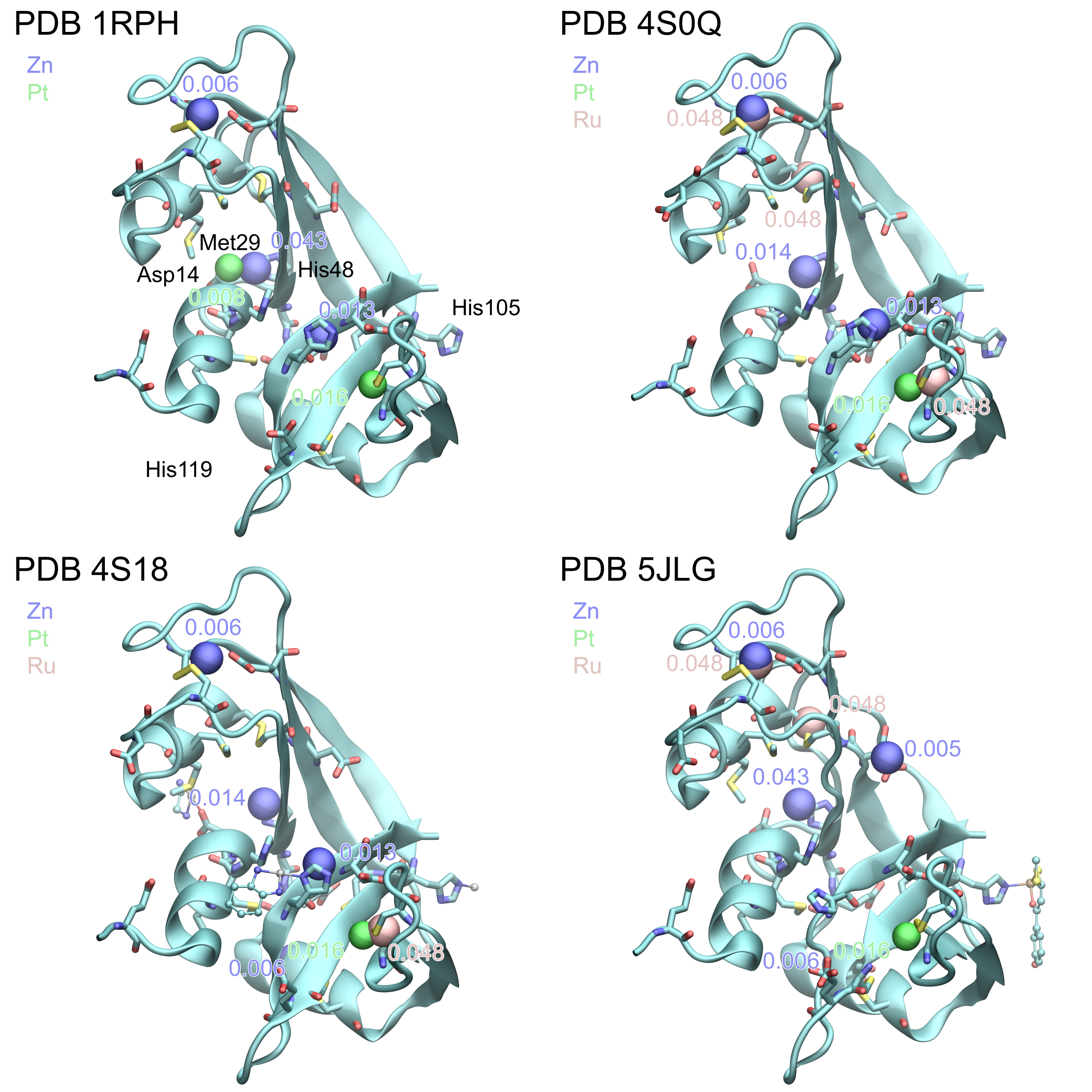

Supplement: S5 Fig — Zinc (blue), platinum (green), and ruthenium (pink). For each prediction, the probability associated with each site is indicated with the same color as the corresponding probability map. (PNG) [file pone.0349622.s010.png]

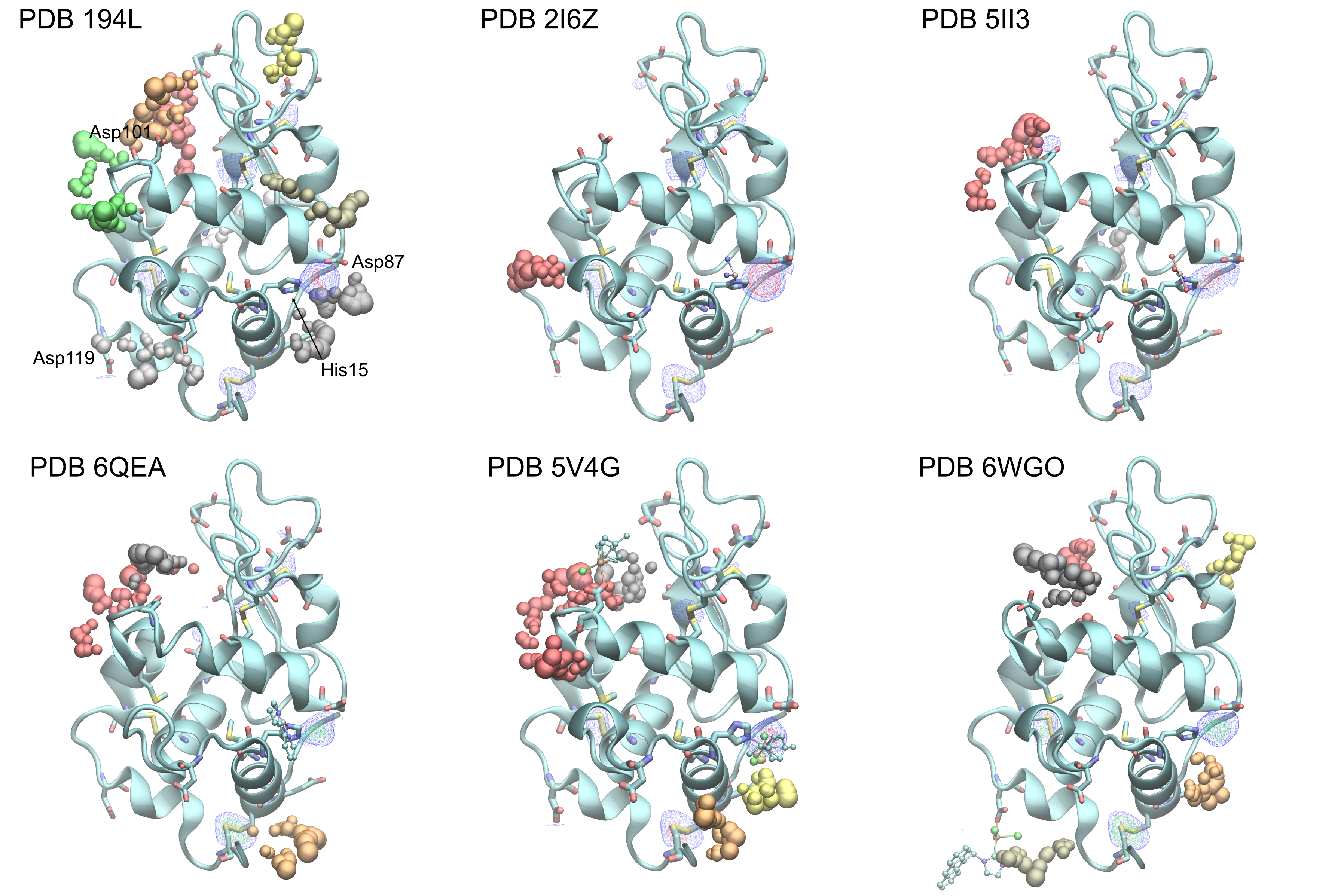

Supplement: S6 Fig — For each prediction, alpha spheres are represented as colored spheres. Metal3D predictions with low (blue) and high (red) probability are also represented as isosurfaces with wireframe representation. (PNG) [file pone.0349622.s011.png]

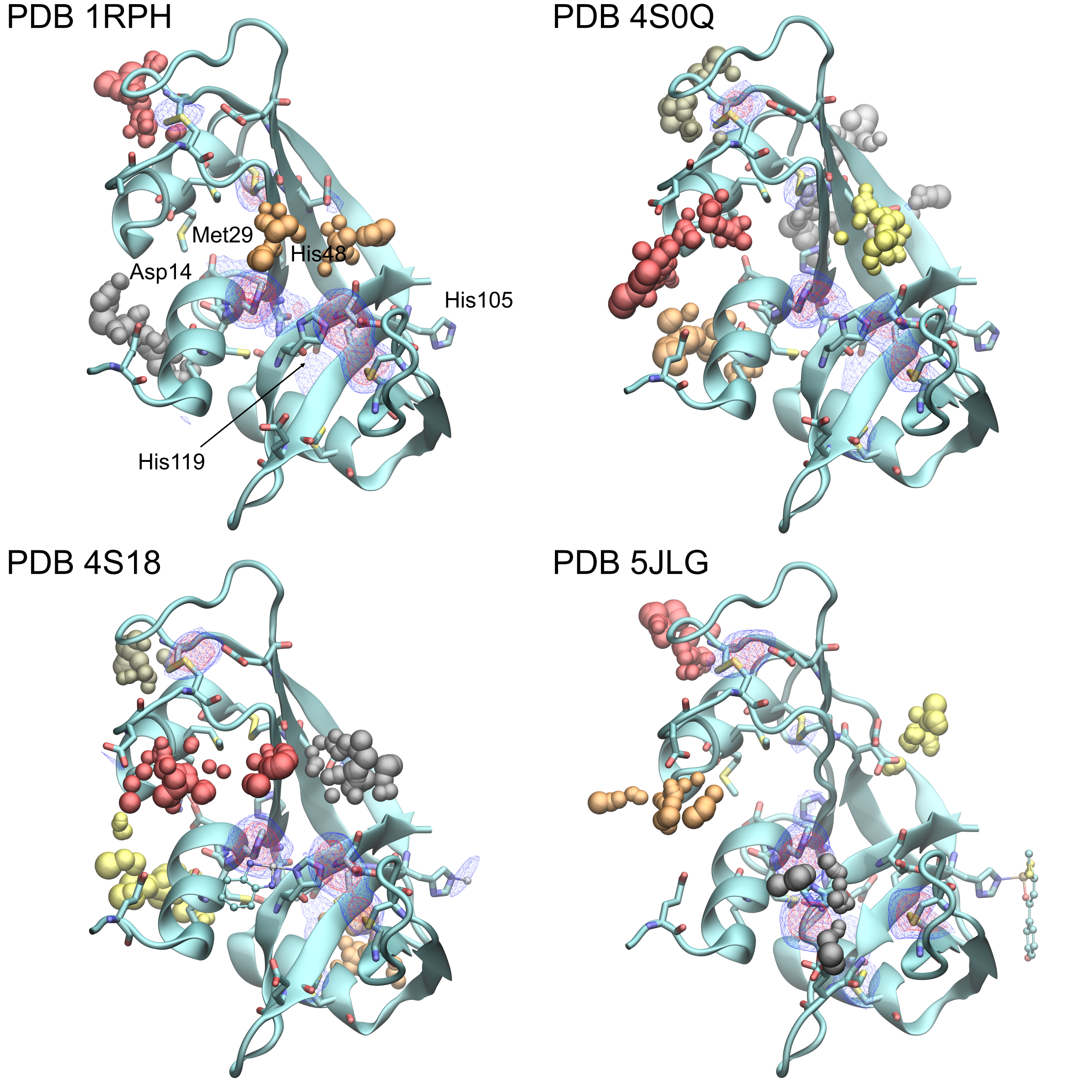

Supplement: S7 Fig — For each prediction, alpha spheres are represented as colored spheres. Metal3D predictions with low (blue) and high (red) probability are also represented as isosurfaces with wireframe representation. (PNG) [file pone.0349622.s012.png]

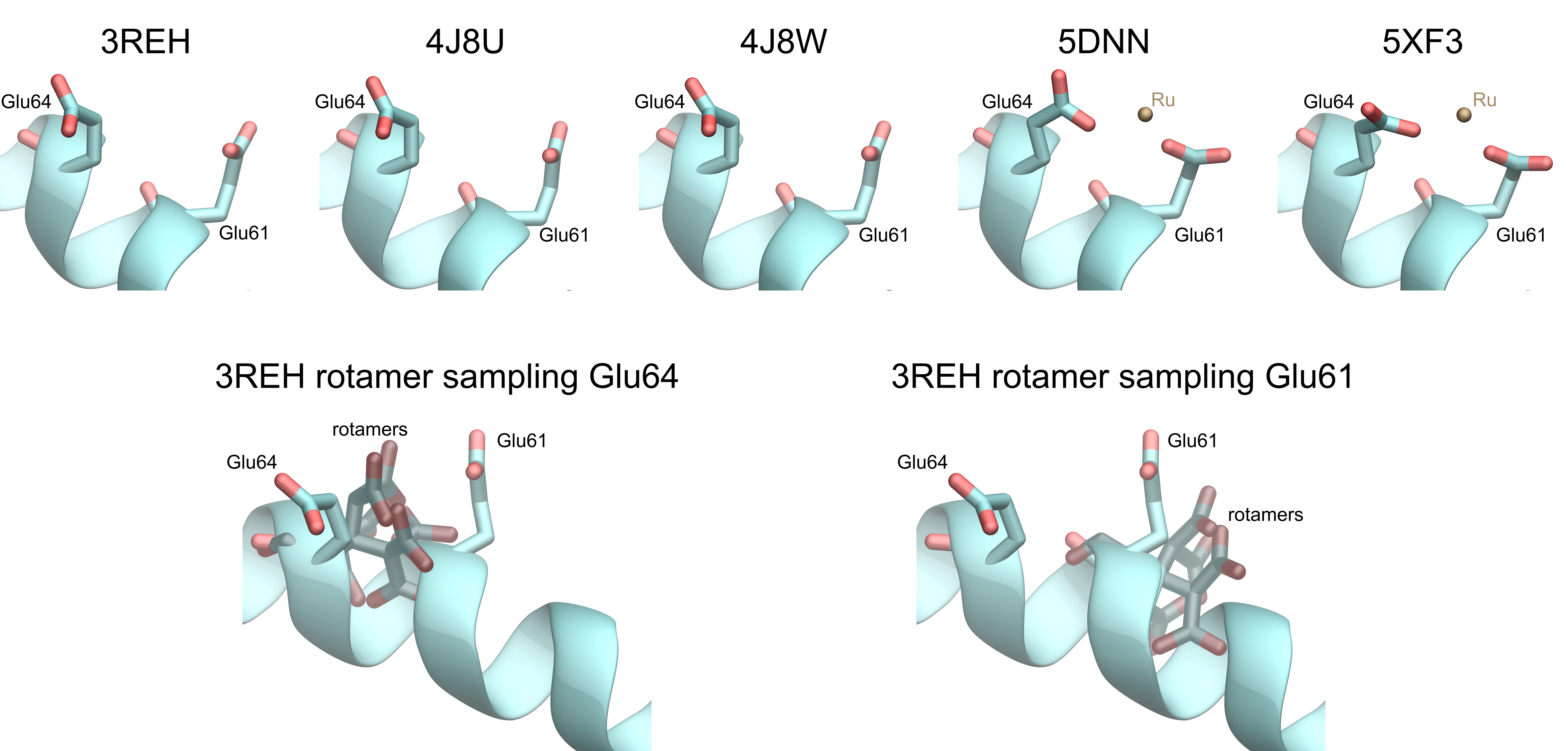

Supplement: S8 Fig — For the apo structure (PDB ID 3REH), the different sampled rotamers for Glu61 and Glu61 are also reported. (PNG) [file pone.0349622.s013.png]
