# Supplementary material for: Predicting unknown binding sites for transition-metal-based compounds in proteins
Source: PLoS One. 2026 Jun 9;21(6):e0349622. doi: 10.1371/journal.pone.0349622 (PMC13249218; doi:10.1371/journal.pone.0349622)
Supplement: Attachment — Submitted filename: review.pdf [file pone.0349622.s014.pdf]

I have carefully read this manuscript, including all figures and the Supporting Information. The work is well-motivated and timely. Applying existing zinc-ion-binding-site predictors (Metal3D and Metal1D) to the harder problem of locating binding sites for covalently bound transition-metal (TM) complexes is a natural and scientifically interesting extension. The demonstration across hen egg-white lysozyme C (HEWLC), bovine pancreatic ribonuclease A (RNaseA), and a bidentate glutamate site in a nucleosome core particle provides a useful proof-of-concept, and the data deposition on Zenodo is commendable. However, after close reading, I identified four categories of concern that, taken together, prevent me from recommending acceptance in the current form. First, the manuscript contains several grammatical and stylistic issues that impede scientific clarity. Second, the validation framework has several significant gaps, as no precision-recall summary table is provided, no baseline comparison with established cavity-detection tools is included, the selection of probability thresholds is never rigorously justified, and no uncertainty is reported for any predicted score. Third, the citation list is incomplete, missing key references for fpocket, structure validation tools, and backbone-dependent rotamer libraries. Fourth, critical reproducibility information is missing, including software version numbers and PDB preprocessing details. I have outlined my concerns below, and I hope the authors find the detailed feedback constructive.

1 Grammar and language

I had difficulty understanding several passages throughout the manuscript. Scientific writing must be precise and unambiguous, and unclear phrasing forces the reader to guess the intended meaning. I urge the authors to carefully revise the English throughout and to rewrite the sentences listed below.

Abstract

1. “Transition metal based compounds are promising therapeutic agents”. Here, throughout the abstract and main text, the compound modifier should be hyphenated as *transition-metal-based compounds*. This applies consistently throughout the manuscript.

2. “predicting their binding sites remains a major challenge”. Here, the antecedent of “their” is ambiguous, and it could refer to the compounds or to the therapeutic agents. I suggest rewriting as: ”Predicting the binding sites of these compounds remains a major challenge.”

3. “both predictors successfully identify several experimentally observed binding sites”. Here, “successfully” is vague and should be replaced by a quantitative word, for example, “correctly identify at least one experimentally observed binding site in each tested protein.” The word “several” should likewise be replaced by the actual number.

Introduction

4. “TM-based drugs often show severe side effects. This is the case for cisplatin, which, despite its several toxic side effects” , Here, the word “several” appears twice in close succession. I suggest removing “its several” and writing “despite its well-known toxic side effects.”

5. “making them impractical to use for high-throughput studies in the case where the binding site for a TM-based compound in a protein is unknown” . This sentence is unnecessarily long. I suggest splitting into two as, ”This makes them impractical for high-throughput binding-site screening, where many putative sites must be assessed.”

6. “Metal3D is able to accurately locate different TM ions” . Here, “different” means “other” (i.e., non-zinc TM ions). Metal3D is able to accurately locate various TM ions beyond zinc.

Methods

7. “to generate a probability density for metal ion location at each grid point starting from the coordinates of the atoms in the input box” . Here, the phrase “starting from” is informal. I suggest rewriting as “to generate a probability density for metal ion location at each grid point, computed from the atomic coordinates within the input box.”

8. “we set a threshold of 0.99, i.e., all predictions with a probability within 99% of the highest-scored one are considered” . This sentence is confusing because 0.99 reads as an absolute probability value, yet it is being used as a relative cutoff. I suggest rewriting as, “we retained all sites whose score was at least 1% of the maximum site score (relative threshold of 0.99).”

9. “The new probability maps, as well as a Jupyter notebook to generate them, have been deposited on Zenodo” . I suggest rewriting as, “We deposited the new probability maps and a Jupyter notebook for their generation at `https://zenodo.org/records/18416988`.”

Results and Discussion

10. “a surprisingly degree of predictive power” . Here,“a surprisingly” is grammatically incorrect. It should be “a surprising degree.”

11. “enabling the identification of the most common binding sites from the apo conformations” . Here, “most common” implies frequency across many proteins, but the authors tested only two enzymes and one NCP system. I suggest “the primary binding sites” instead.

12. “This is particularly evident in the case of Pt-based compounds, where methionine binding becomes preferential” . Here, the verb “becomes” is inappropriate because the preference is a static property of the training dataset, not a change over time. I suggest “is preferential.”

13. “the probability associated with each site is reported in the figure”. Here, this phrase appears in multiple figure captions. “Reported” should be “indicated” or “labeled,” since the values appear as annotations in the figure rather than in a separate report.

Conclusion

14. “Metal3D and Metal1D can serve as a first step in establishing rapid, low-cost pre-screening tools”. Here, “a first step in establishing” is redundant because the authors have already demonstrated this in the paper. I suggest rewriting to, “Metal3D and Metal1D represent rapid, low-cost pre-screening tools.”

15. “Both approaches pose non-trivial challenges, as in Monte Carlo, suitable trial moves need to be established, balancing the increase in the predictor probability with maintaining energetically favorable conformations” . This is a run-on sentence. I suggest splitting after “need to be established.” and beginning the second sentence with “These moves must balance. . . ”

2 Technical concerns

I find the following methodological issues sufficiently serious to require explicit discussion or correction in the revised manuscript.

1. The lower isovalue of $p \approx 0.04$ is used throughout the manuscript to "locate distinct sites" with Metal3D, yet this threshold is never rigorously justified. I would like to know whether it is chosen empirically to match the visual appearance of the isosurface, or whether it corresponds to a physically meaningful cutoff. For instance, the value at which Metal3D achieves a specified precision on its original validation set. I ask the authors to either provide a statistical justification (e.g., a precision-recall curve as a function of isovalue on the Metal3D validation set) or explicitly acknowledge that this threshold is heuristic and discuss its sensitivity to the reported conclusions.

2. The entire manuscript rests on the hypothesis that a model trained exclusively on zinc ion coordination environments generalises to Pt- and Ru-based organometallic complexes. Zinc typically forms tetrahedral or octahedral coordination shells with His, Cys, Asp, and Glu, whereas the compounds studied here form monodentate or bidentate adducts using planar or piano-stool geometries. I think the authors should provide a more rigorous justification for this domain transfer. What fraction of the Metal3D training environments are geometrically compatible with a monodentate TM complex? I would like the authors to provide a quantitative analysis of training-set overlap in the coordination-geometry space, or, at a minimum, a detailed discussion of why the transfer is expected to be valid despite these geometric differences.

3. Metal3D operates on a 16 Å box centered on a Cα atom and encodes channels including occupancy and hydrophobicity, but the organic ligands coordinating the Pt or Ru center are entirely absent from the input. The steric and electronic perturbation introduced by, for example, the cymene cap in the Ru(II)(cymene) complexes (PDB 5V4G, 6WGO) could either occlude or expose a binding site relative to the bare-protein prediction. I find this a fundamental limitation that is only briefly mentioned in the Conclusion. I would like the authors to add a dedicated paragraph in the Limitations section that estimates the size of the excluded volume of the organic ligands relative to the 16 Å input box, and to discuss whether this omission could lead to systematic false negatives for sterically demanding compounds.

4. The authors describe prediction success and failure for individual sites in narrative form, but I cannot find a single summary table reporting precision and recall (or sensitivity and specificity) for each predictor, each probability map, and each protein system. Without such a table, it is impossible to compare Metal3D versus Metal1D, or the zinc versus Pt versus Ru probability maps, in a statistically meaningful way. I ask the authors to add a table in the main text or SI reporting for each (predictor, probability map, protein) combination, showing the numbers of true positive, false positive, and false negative site predictions at the thresholds used.

5. The manuscript consistently reports which known binding sites are or are not predicted (sensitivity), but never reports how many predicted sites do not correspond to any known TM binding site (false positive rate). For HEWLC, the disulfide bridges are identified as false positives in passing, but their count is not quantified, and in RNaseA, there are additional predicted regions that are not discussed. I think the authors should report the total number

of predicted sites per protein, per predictor, and per threshold, partitioned into true positives and false positives, so that the practical utility of the tools can be objectively evaluated.

6. Both Metal3D and Metal1D consistently predict the disulfide bridge regions as putative TM binding sites in both HEWLC and RNaseA. The authors note that these can be discarded "by visual inspection," but I think this requires more careful treatment. In a real drug discovery pipeline, how would a user know a priori which predicted sites correspond to disulfide bridges? Would a simple post-processing rule, for instance, excluding all predicted sites within 4 Å of any Cys–Cys bridge, eliminate these false positives without removing true positives? I would like the authors to evaluate this filtering step and report its effect on precision and recall.

7. The rotamer sampling follows the Richardson penultimate rotamer library, which represents backbone-independent torsion angle distributions. I find two concerns here. First, for amino acids in constrained binding pocket environments, backbone-dependent rotamer probabilities (e.g., the Dunbrack library) may differ substantially. Have the authors assessed whether backbone-dependent rotamers would change the outcome for His15 or His105? Second, after generating each rotamer, no energy minimization or even steric clash check is performed. Some sampled rotamers may introduce severe van der Waals clashes with neighbouring residues, rendering them physically irrelevant. I would like the authors to either add a steric screening step or demonstrate that none of the rotamers used have MolProbity clash scores above a reasonable threshold (e.g., 40).

8. The Ru-based probability map is derived from only 28 unique protein structures containing 47 Ru binding sites. The original Metal1D zinc map used approximately 2,085 structures with roughly 15,600 binding sites, a factor of $\sim$75 more data than the original. I find it surprising that the authors present the Ru map as a usable predictive tool, given the sparse data. I would like the authors to provide a statistical analysis of the reliability of the Ru map. For example, a bootstrap or leave-one-out cross-validation demonstrates that the residue binding frequencies in Fig. 2 are stable and not an artefact of the small sample size. If the map is not statistically reliable, the authors should state this explicitly and caution against its use.

9. Throughout the manuscript, predicted probabilities from Metal3D are reported as single point values (e.g., $p \sim 0.14$, $p \sim 0.04$, $p \sim 0.2$), and Metal1D scores are reported to three decimal places (e.g., 0.032, 0.008). No uncertainty or variance is reported for any of these values. For Metal3D, the prediction is generated by averaging probability boxes across different Cα-centred windows, so in principle a variance can be computed. For Metal1D, the score depends on a geometric search that is sensitive to small coordinate perturbations. I think the authors should report either confidence intervals or a measure of score sensitivity to small coordinate perturbations (e.g., ± 0.1 Å RMSD noise on the input structure).

10. All conclusions about whether a site is "predicted" or "not predicted" by Metal3D rest on the choice of isovalue thresholds (0.04 for the lower contour; 0.14 or 0.16 for the higher contour). I would like the authors to perform a sensitivity analysis of the effects on precision and recall as the lower threshold is varied from 0.02 to 0.08. Is the qualitative conclusion that His15 is predicted but Asp101 and Asp119 are not robust across this range, or is it threshold-dependent? For Metal1D, an analogous analysis varying the relative threshold from 0.95 to 0.999 should be provided.

11. Metal1D scores each putative site by summing probabilities from its LINK-derived probability map over all compatible coordination environments within the search radius. I want to under-

stand more clearly what physical quantity this score approximates. The LINK records in the PDB reflect which coordination environments happen to have been crystallised and deposited, and are therefore biased by crystallisation conditions, resolution limits, and the popularity of particular protein families as model systems. The score is not a binding free energy, a structural stability measure, or a thermodynamic quantity. Does this purely frequency-based score have predictive validity for identifying novel TM binding sites in proteins not represented in the training set? I think a more careful discussion of the statistical nature and limitations of the Metal1D scoring function is needed, supported by a leave-one-protein-out validation.

12. The manuscript benchmarks Metal3D and Metal1D against each other and against the known experimental binding sites, but never compares them against any established binding site detection tool such as fpocket, SiteMap (Schrödinger), or CASTp. I find this lack of baseline comparators a significant weakness: it is possible that a generic cavity-detection algorithm would achieve similar or better recall on the tested systems, since all tested binding sites involve surface-exposed or shallow clefts. I ask the authors to include at least one publicly available baseline (FPocket is free and widely used) and to report its precision and recall on the same test set under the same evaluation criteria.

13. Throughout the paper, a predicted site is considered a "hit" if a Metal3D probability region or a Metal1D coordinate is visually in proximity to the known binding amino acid. I find this criterion vague and inconsistently applied. What is the exact distance threshold used to call a prediction a true positive, that is, how close (in Å) must the predicted site centroid be to the known binding atom? Is this criterion identical for all three protein systems? I ask the authors to state this threshold explicitly and apply it uniformly.

14. Predictions are primarily presented for apo structures, but the resolution of PDB 194L (the primary HEWLC apo reference) is not reported anywhere in the main text or in the SI RMSD tables. Since Metal3D uses atomic coordinates directly and the quality of side-chain orientations depends on resolution, I ask the authors to report the resolution of each PDB structure used in Tables S1 and S2 and to discuss whether any low-resolution structures may have introduced coordinate uncertainty that affects the predictions.

3 Literature review and data reproducibility

I find the following citation issues significant enough to require correction before the manuscript can be considered complete. The data availability for this manuscript is better than average, as the authors have deposited predictions, visualisations, and a Jupyter notebook on Zenodo, which I commend. Nevertheless, I find several specific reproducibility gaps that must be addressed.

1. Reference [33] (Levy et al., bioRxiv 2025, doi:10.1101/2025.11.13.688318) is cited in the Methods as the primary source for the NCP GLU site test system. Since one of the authors of the current manuscript is also an author on Ref. [33], I ask the authors to confirm whether a peer-reviewed journal version is now available, and if so, to update the citation. Citing one's own unpublished preprint as the primary source of experimental data for a test system raises concerns about reproducibility.

2. No comparison is made with established binding site predictors, and the relevant literature is not cited. If the authors choose not to include a numerical baseline comparison, they should at a minimum cite fpocket, SiteMap, and CASTp, and explicitly discuss why Metal3D and Metal1D are expected to outperform these tools for TM binding sites.

3. The manuscript cites the Richardson penultimate rotamer library [44] for rotamer sampling. Since backbone-dependent rotamers are a relevant methodological consideration, the Dunbrack & Cohen backbone-dependent rotamer library should also be cited and its non-use justified.

4. No structure validation is reported for the PDB structures used as input. I ask the authors to cite MolProbity or an equivalent tool and to confirm that all structures used have acceptable geometry (Ramachandran outliers below 2%, rotamer outliers below 5%, clashscore below 20). This is particularly important for PDB 3REH and 5XF3, which are the sole basis for the bidentate GLU site analysis.

5. Reference [43] (EVOLVE, ChemRxiv 2023) is cited for the rotamer sampling approach used in this work. I would like the authors to verify whether a peer-reviewed version of the EVOLVE paper is now available and, if so, to update the citation. Methodological choices should ideally be grounded in peer-reviewed sources.

6. The probability map generation from LINK records is described only briefly and deferred entirely to Ref. [12]. However, since the authors generate two new maps (Pt and Ru) in this paper using the same approach, and since the small dataset size raises questions about statistical validity, I think a brief self-contained description of the map generation procedure, including how LINK records were filtered, how duplicate structures were removed, and how probabilities were normalised and should be provided in the Methods or SI. This information is necessary for reproducibility.

7. The manuscript does not report version numbers for Metal3D, Metal1D, or VMD. Since both predictors are under active development, results may differ across versions. I ask the authors to report the exact versions of all software tools used, including the Metal3D/Metal1D package and the VMD versions, in the Methods section.

8. The preprocessing steps applied to raw PDB structures before running the predictors are not described anywhere. Specifically, (a) were hydrogen atoms added, and if so, with which tool and at what pH, (b) were alternate conformations resolved, and if so, how, (c) were crystallographic water molecules and co-crystallised ligands removed from the input file or merely ignored by the code? The manuscript states that "non-protein atoms are not considered," but it is not clear whether they were physically removed from the input file. This distinction matters because some implementations may behave differently depending on whether extraneous atoms are present.

9. I ask the authors to confirm explicitly in the Methods which files are deposited on Zenodo, including (a) the probability map files for Pt and Ru in their native format, (b) the input PDB files as actually used after preprocessing, (c) the Metal3D global probability density files (.dx or .cube format) for all structures shown in the figures, and (d) the rotamer-sampled PDB coordinate files for the structures shown in Figs. 7 and 8. Without these files, an independent researcher cannot reproduce Figs. 4, 6, 7, and 8.

10. The rotamer sampling procedure is described as generating conformations from the Richardson library, but no random seed is reported. If the sampling involves any stochastic element (e.g., random selection among rotamers of similar probability), results would not be exactly reproducible without a fixed seed. I ask the authors to clarify whether the sampling is entirely deterministic or stochastic, and if stochastic, to report the random seed used.

11. The exact Metal1D search radius used in this study is not reported. The original Ref. [12] describes a default value, but since the organic ligands coordinating the TM center impose additional steric constraints, I want to confirm whether the default search radius was used or adjusted for TM complexes. This parameter must be stated explicitly in the Methods.
