# Supplementary material for: Predicting unknown binding sites for transition-metal-based compounds in proteins
Source: PLoS One. 2026 Jun 9;21(6):e0349622. doi: 10.1371/journal.pone.0349622 (PMC13249218; doi:10.1371/journal.pone.0349622)
Supplement: Attachment — Submitted filename: response_reviewers.pdf [file pone.0349622.s016.pdf]

Response to Reviewer Comments

Predicting unknown binding sites for transition-metal-based compounds in proteins

Reviewer 1

I have carefully read this manuscript, including all figures and the Supporting Information. The work is well-motivated and timely. Applying existing zinc-ion-binding-site predictors (Metal3D and Metal1D) to the harder problem of locating binding sites for covalently bound transition-metal (TM) complexes is a natural and scientifically interesting extension. The demonstration across hen egg-white lysozyme C (HEWLC), bovine pancreatic ribonuclease A (RNaseA), and a bidentate glutamate site in a nucleosome core particle provides a useful proof-of-concept, and the data deposition on Zenodo is commendable. However, after close reading, I identified four categories of concern that, taken together, prevent me from recommending acceptance in the current form. First, the manuscript contains several grammatical and stylistic issues that impede scientific clarity. Second, the validation framework has several significant gaps, as no precision-recall summary table is provided, no baseline comparison with established cavity-detection tools is included, the selection of probability thresholds is never rigorously justified, and no uncertainty is reported for any predicted score. Third, the citation list is incomplete, missing key references for fpocket, structure validation tools, and backbone-dependent rotamer libraries. Fourth, critical reproducibility information is missing, including software version numbers and PDB preprocessing details. I have outlined my concerns below, and I hope the authors find the detailed feedback constructive.

We thank the reviewer for the positive comments and for the detailed feedback that they provided. Based on these, we made substantial modifications to the manuscript to clarify the text and strengthen the results, in particular by adding statistical considerations and performing a comparison with an additional prediction tool (Fpocket) to provide a comparison of Metal1D and Metal3D with a cavity-detection algorithm.

The data related to this article, including the additions made to address the reviewer's comments, are collected in the same Zenodo repository as the original submission (https://zenodo.org/records/18416988). The repository will be made publicly accessible upon acceptance and has already been made available to reviewers during the peer-review process via this link (Version v2):
https://zenodo.org/records/18416989?token=eyJhbGciOiJIUzUxMiJ9.eyJpZCI6IjE2YTMzYmJlLTkxNjMtNDViZC1iYjZjLWI1NTkyN2I0OTQwOSIsImRhdGEiOnt9LCJyYW5kb20iOiIyMmZkMDU4ZDc0Njg0MjcyNGQxZTlhMjIyNTQ0ODUyNyJ9.31EuNAod7g8_exy8vb3azuveJkSYBUL5OehCIqyfNje98LBusVuDARRzq3BzTWh5RcN4kh9kW7fpN55mOOBdRQ

Grammar and language: I had difficulty understanding several passages throughout the manuscript. Scientific writing must be precise and unambiguous, and unclear phrasing

forces the reader to guess the intended meaning. I urge the authors to carefully revise the English throughout and to rewrite the sentences listed below.

1. Transition metal based compounds are promising therapeutic agents”. Here, throughout the abstract and main text, the compound modifier should be hyphenated as transition-metal-based compounds. This applies consistently throughout the manuscript.
   We adapted the hyphenation.

2. “predicting their binding sites remains a major challenge”. Here, the antecedent of “their” is ambiguous, and it could refer to the compounds or to the therapeutic agents. I suggest rewriting as: ”Predicting the binding sites of these compounds remains a major challenge.”
   We thank the reviewer for highlighting this potential source of confusion, and we changed the sentence to “predicting the binding sites of such compounds remains a major challenge”.

3. “both predictors successfully identify several experimentally observed binding sites”. Here, “successfully” is vague and should be replaced by a quantitative word, for example, “correctly identify at least one experimentally observed binding site in each tested protein.” The word “several” should likewise be replaced by the actual number.
   We agree with this comment, and we rephrased the sentence in order to be more precise. Now it reads “both predictors correctly identify at least one of the experimentally observed binding sites for transition metal complexes in each of the apo protein structures tested”.

4. “TM-based drugs often show severe side effects. This is the case for cisplatin, which, despite its several toxic side effects” , Here, the word “several” appears twice in close succession. I suggest removing “its several” and writing “despite its well-known toxic side effects.”
   In the sentence highlighted, there is no close succession of the word “several”. This observation might come from the nearby word “severe”, referring to cisplatin. We decided to keep the text unchanged.

5. “making them impractical to use for high-throughput studies in the case where the binding site for a TM-based compound in a protein is unknown” . This sentence is unnecessarily long. I suggest splitting into two as, ”This makes them impractical for high-throughput binding-site screening, where many putative sites must be assessed.”
   We thank the reviewer for suggesting this modification to improve clarity. We rephrased this sentence, now reading “However, such methods are orders of magnitude more computationally expensive than molecular docking or even classical FFs approaches. This makes them impractical for high-throughput studies, where various putative sites must be assessed.”

6. “Metal3D is able to accurately locate different TM ions” . Here, “different” means “other” (i.e., non-zinc TM ions). Metal3D is able to accurately locate various TM ions beyond zinc.

We rewrote the original sentence to improve clarity. Now the full sentence reads: “Albeit solely trained on zinc, Metal3D is able to accurately locate TM ions different from zinc.”

7. “to generate a probability density for metal ion location at each grid point starting from the coordinates of the atoms in the input box” . Here, the phrase “starting from” is informal. I suggest rewriting as “to generate a probability density for metal ion location at each grid point, computed from the atomic coordinates within the input box.”
   We thank the reviewer for the suggestion. We changed the sentence to the suggested one.

8. “we set a threshold of 0.99, i.e., all predictions with a probability within 99% of the highestscored one are considered” . This sentence is confusing because 0.99 reads as an absolute probability value, yet it is being used as a relative cutoff. I suggest rewriting as, “we retained all sites whose score was at least 1% of the maximum site score (relative threshold of 0.99).”
   We rephrased this sentence, highlighting that 0.99 is a Metal1D-specific threshold value “we retained all sites with a score of at least 1% of the maximum site score (Metal1D threshold parameter of 0.99).

9. “The new probability maps, as well as a Jupyter notebook to generate them, have been deposited on Zenodo” . I suggest rewriting as, “We deposited the new probability maps and a Jupyter notebook for their generation at https://zenodo.org/records/18416988.”
   We agree with the reviewer’s comment and adopted their suggested new version of this sentence.

10. “a surprisingly degree of predictive power” . Here,“a surprisingly” is grammatically incorrect. It should be “a surprising degree.”
    The sentence was already “a surprising degree” in the original version.

11. “enabling the identification of the most common binding sites from the apo conformations” . Here, “most common” implies frequency across many proteins, but the authors tested only two enzymes and one NCP system. I suggest “the primary binding sites” instead.
    We agreed with the reviewer and adopted their suggested new version of the sentence.

12. “This is particularly evident in the case of Pt-based compounds, where methionine binding becomes preferential” . Here, the verb “becomes” is inappropriate because the preference is a static property of the training dataset, not a change over time. I suggest “is preferential.”
    Changed as suggested by the reviewer.

13. “the probability associated with each site is reported in the figure”. Here, this phrase appears in multiple figure captions. “Reported” should be “indicated” or “labeled,” since the values appear as annotations in the figure rather than in a separate report.

We have changed “reported” to “indicated” in Figs. 4, 5, S2, and S5.

14. “Metal3D and Metal1D can serve as a first step in establishing rapid, low-cost pre-screening tools”. Here, “a first step in establishing” is redundant because the authors have already demonstrated this in the paper. I suggest rewriting to, “Metal3D and Metal1D represent rapid, low-cost pre-screening tools.”
    We agree with the reviewer and changed the sentence as suggested.

15. “Both approaches pose non-trivial challenges, as in Monte Carlo, suitable trial moves need to be established, balancing the increase in the predictor probability with maintaining energetically favorable conformations” . This is a run-on sentence. I suggest splitting after “need to be established.” and beginning the second sentence with “These moves must balance. . . ”
    We have changed the sentence to “Both approaches pose non-trivial challenges, as in Monte Carlo, suitable trial moves need to be established. Such moves must balance …”

Technical concerns: I find the following methodological issues sufficiently serious to require explicit discussion or correction in the revised manuscript.

1. The lower isovalue of p ≈ 0.04 is used throughout the manuscript to “locate distinct sites” with Metal3D, yet this threshold is never rigorously justified. I would like to know whether it is chosen empirically to match the visual appearance of the isosurface, or whether it corresponds to a physically meaningful cutoff. For instance, the value at which Metal3D achieves a specified precision on its original validation set. I ask the authors to either provide a statistical justification (e.g., a precision-recall curve as a function of isovalue on the Metal3D validation set) or explicitly acknowledge that this threshold is heuristic and discuss its sensitivity to the reported conclusions.
    As we explained in the Methods section, “we analysed regions with relatively low probability values by finding the minimal isovalue that enabled localizing distinct sites around the protein (p~0.04).” This value has been determined empirically as a value that enables us to localize distinct sites, while for lower isovalues, the probability density is spread across the whole box. For clarification, we provide here some examples with lower isovalues:

p=0.0004 p=0.004 p=0.04

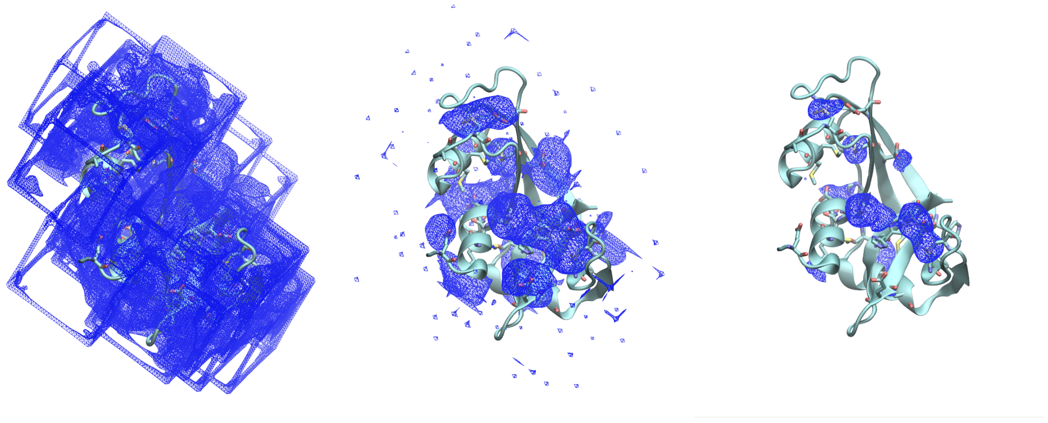

To clarify this, we changed the aforementioned sentence, mentioning that this

parameter was empirically determined by visual inspection, and that it can depend on the system (e.g., we have 0.04 and 0.06 for the two proteins): "... we analysed regions with relatively low probability values. By visual inspection of the isosurfaces of the probability density, we empirically determined a minimal isovalue that enabled localizing distinct sites around each of the proteins tested (p=0.04 for HEWLC and p=0.06 for RNaseA)."

2. The entire manuscript rests on the hypothesis that a model trained exclusively on zinc ion coordination environments generalises to Pt- and Ru-based organometallic complexes. Zinc typically forms tetrahedral or octahedral coordination shells with His, Cys, Asp, and Glu, whereas the compounds studied here form monodentate or bidentate adducts using planar or piano-stool geometries. I think the authors should provide a more rigorous justification for this domain transfer. What fraction of the Metal3D training environments are geometrically compatible with a monodentate TM complex? I would like the authors to provide a quantitative analysis of training-set overlap in the coordination-geometry space, or, at a minimum, a detailed discussion of why the transfer is expected to be valid despite these geometric differences.
   In the original Metal3D/1D work, the details of the dataset are analysed extensively, including the coordination motifs. Even if zinc typically forms tetrahedral or octahedral coordination shells, the dataset is more varied, also presenting di- or even mono-coordinated geometries. We added the following sentence to point the reader to the original publication, also for the details on the dataset "A detailed description of Metal3D and Metal1D can be found in the original publication by Dürr et al. In the same work, the dataset composition is analysed, including the distribution of coordination motifs."

   The transferability of a model trained on Zn ions to TM ions was tested in the original publication, demonstrating that although sites with more than 3 binding amino acids were clearly predicted with higher accuracy, both Metal1D and Metal3D showed prediction ability also for di- and mono-dentate sites. This surprising ability is "stress-tested" under even more different conditions in the current work, where we investigate TM-based compounds.

3. Metal3D operates on a 16 Å box centered on a Cα atom and encodes channels including occupancy and hydrophobicity, but the organic ligands coordinating the Pt or Ru center are entirely absent from the input. The steric and electronic perturbation introduced by, for example, the cymene cap in the Ru(II)(cymene) complexes (PDB 5V4G, 6WGO) could either occlude or expose a binding site relative to the bare-protein prediction. I find this a fundamental limitation that is only briefly mentioned in the Conclusion. I would like the authors to add a dedicated paragraph in the Limitations section that estimates the size of the excluded volume of the organic ligands relative to the 16 Å input box, and to discuss whether this omission could lead to systematic false negatives for sterically demanding compounds.
   We thank the reviewer for raising this important point. In the current implementation of Metal3D used in this study, the model is trained on single Zn ions with a 16Å cubic box. This box size was optimized for the prediction of bare metal ions in the original Metal3D work. As pointed out by the reviewer, when considering TM complexes with organic ligands coordinating the metal center, the steric volume may extend beyond

this box. In such cases, when training the 3DCNN on the TM complexes, the box size would likely need to be adapted, based on the specific dataset.
The focus of the present work is to evaluate whether a predictor trained on metal ions can provide useful information for the location of TM-based compound binding "hotspots". We do not attempt to retrain the model on TM compounds and optimize the box size. We now explicitly mention this limitation in the conclusions and highlight the possibility of increasing the box size.

The relevant sentence now reads:
"This could be taken into account, e.g., in Metal3D by retraining the 3DCNN on datasets of TM complexes to properly capture the influence of the coordinating ligands, although the limited availability of such structures currently represents a major limitation to this strategy. For this purpose, the size of the boxes centered on the Cα atoms may need to be increased, as the 16Å cubic box used for zinc ions might be too small to fully capture the steric environment of the coordinating ligands."

4. The authors describe prediction success and failure for individual sites in narrative form, but I cannot find a single summary table reporting precision and recall (or sensitivity and specificity) for each predictor, each probability map, and each protein system. Without such a table, it is impossible to compare Metal3D versus Metal1D, or the zinc versus Pt versus Ru probability maps, in a statistically meaningful way. I ask the authors to add a table in the main text or SI reporting for each (predictor, probability map, protein) combination, showing the numbers of true positive, false positive, and false negative site predictions at the thresholds used.
We thank the reviewer for raising this important point. Due to the limited number of experimentally available structures, we believe that a fully quantitative statistical comparison (precision/recall for all predicted sites) is not meaningful. In particular, classifying a prediction as a false positive can be misleading, as unobserved sites in a limited number of structures may still be relevant and the results of this comparison might change significantly (e.g., for the RNaseA case, His119 is only occupied in one structure out of the four considered).

To provide a fair and informative comparison, we report precision and recall only for sites with experimental confirmation of metal occupancy by at least one of the structures, where true positives and false negatives can be clearly defined. This implies that only recall values can be computed, as for a proper assessment of precision/recall we would need a large dataset with clearly defined TP/FP/FN. For this analysis, we considered all structures investigated, defining a prediction as TP if a predicted location is within 5 Å of an observed metal center, and for Metal3D if a probability density region (with different thresholds, to show the sensitivity to this parameter) is within the same range.

We have added a summary table in the SI, showing the recall for each site, and referred to it in the discussion of the results.

Table S1: Summary of the true positive (TP), false negative (FN), and recall values for the different predictors and probability thresholds/maps. For each case, the values reported are: TP/FN (recall). For Metal3D, the thresholds for p(low), p(medium), and p(high) are 0.04, 0.09, and 0.14 for HEWLC, and 0.06, 0.11, and 0.16 for RNaseA.

		Metal3D			Metal1D		
Protein	Site	p(high)	p(medium)	p(low)	Zn map	Pt map	Ru map
HEWLC	His15	4/2 (0.66)	6/0 (1.0)	6/0 (1.0)	5/1 (0.83)	5/1 (0.83)	0/6 (0.0)
	Asp101	0/6 (0.0)	0/6 (0.0)	3/3 (0.50)	0/6 (0.0)	0/6 (0.0)	0/6 (0.0)
	Asp119	0/6 (0.0)	0/6 (0.0)	3/3 (0.50)	0/6 (0.0)	0/6 (0.0)	0/6 (0.0)
RNaseA	His119	4/0 (1.0)	4/0 (1.0)	4/0 (1.0)	3/1 (0.75)	0/4 (0.0)	0/4 (0.0)
	His105	0/4 (0.0)	1/3 (0.25)	1/3 (0.25)	0/4 (0.0)	0/4 (0.0)	0/4 (0.0)
	Asp14	0/4 (0.0)	0/4 (0.0)	1/3 (0.25)	0/4 (0.0)	1/3 (0.25)	0/4 (0.0)

To emphasize the context and limitations of this comparison, we added the following paragraph in the Methods section of the manuscript:
"Due to the limited number of available protein structures in our dataset, a fully quantitative statistical comparison is not feasible. In particular, classifying a prediction as a false positive (FP) can be misleading, because unobserved sites in this small number of crystal structures may still be biologically relevant or occupied by a different TM complex. In contrast, true positives (TPs) and false negatives (FNs) can be defined for the experimentally known sites in our dataset, and hence the recall, defined as TP/(TP+FN). The results of this analysis are reported in Table S1. To provide a meaningful comparison, in the case of Metal1D, we classified a prediction as TP or FN if it was located within 5Å of the experimentally observed metal center in at least one of the TM complexes. For Metal3D, the same procedure was applied to probability density regions within 5Å of an experimental metal center, testing three different probability thresholds per protein based on the minimum isovalue identified for the system. Specifically, we used low, medium, and high thresholds of p>=0.04, 0.09, and 0.14 for HEWLC, and 0.06, 0.11, and 0.16 for RNaseA. This approach allows a fair comparison of the different predictors while acknowledging the limitations of the dataset and the uncertainty associated with unobserved sites."

5. The manuscript consistently reports which known binding sites are or are not predicted (sensitivity), but never reports how many predicted sites do not correspond to any known TM binding site (false positive rate). For HEWLC, the disulfide bridges are identified as false positives in passing, but their count is not quantified, and in RNaseA, there are additional predicted regions that are not discussed. I think the authors should report the total number of predicted sites per protein, per predictor, and per threshold, partitioned into true positives and false positives, so that the practical utility of the tools can be objectively evaluated.
As discussed in the reply to the previous question, in the cases tested, the definition of FP is not clear and statistically meaningful. With the additions mentioned in the response to the previous question, and the additional paragraph commenting on this specific aspect, we think that this choice should now be well-justified to the reader.

6. Both Metal3D and Metal1D consistently predict the disulfide bridge regions as putative TM binding sites in both HEWLC and RNaseA. The authors note that these can be discarded “by visual inspection,” but I think this requires more careful treatment. In a real drug discovery pipeline, how would a user know a priori which predicted sites correspond to disulfide bridges? Would a simple post-processing rule, for instance, excluding all predicted sites within 4 Å of any Cys–Cys bridge, eliminate these false positives without removing true positives? I would like the authors to evaluate this filtering step and report its effect on precision and recall.
   We thank the reviewer for raising this point concerning the predictions of disulfide bridges. Concerning the identification of disulfide bridges, we agree that the statement about “visual inspection” may be an oversimplification. In practice, such regions can be identified automatically using simple structural criteria or by making use of the “SSBOND” sections in PDB files.

   We have therefore clarified this point in the manuscript and suggested a practical strategy to flag predictions located near cys-cys disulfide bonds:
   “In practice, when investigating binding sites for TMs compounds, predictions associated with disulfide bridges can be identified by visual inspection or automatically flagged in a post-processing step detecting cysteine pairs forming disulfide bonds, e.g., by geometrical criteria or by using the information contained in the SSBOND section of PDB files. Predicted sites located within a short distance of the corresponding sulfur atoms can therefore be excluded. For example, applying this criterion to all Metal1D predictions shows that none of the putative sites in the proximity of a disulfide bridge would be retained when discarding sites located within 3.0 Å of the sulfur atoms involved in the disulfide bond (maximum distance for the predictions 2.87 Å).”

7. The rotamer sampling follows the Richardson penultimate rotamer library, which represents backbone-independent torsion angle distributions. I find two concerns here. First, for amino acids in constrained binding pocket environments, backbone-dependent rotamer probabilities (e.g., the Dunbrack library) may differ substantially. Have the authors assessed whether backbone-dependent rotamers would change the outcome for His15 or His105? Second, after generating each rotamer, no energy minimization or even steric clash check is performed. Some sampled rotamers may introduce severe van der Waals clashes with neighbouring residues, rendering them physically irrelevant. I would like the authors to either add a steric screening step or demonstrate that none of the rotamers used have MolProbity clash scores above a reasonable threshold (e.g., 40).
   We thank the reviewer for raising this point. The main goal of the rotamer sampling performed in this study was not to identify the most favorable rotameric state for a given residue, but rather to illustrate the potential influence of side chain orientation on the predicted sites. Indeed, as shown in the tested cases, changing the rotameric state can significantly affect the predicted probability regions.

   At this stage, the rotamers were generated only to explore the geometric sensitivity of the predictors in the case of TM compounds, without attempting to identify the physically optimal conformations. As correctly noted by the reviewer, a realistic assessment would require additional considerations, such as steric clashes, local

relaxations through energy minimization, but also considering the free energy cost associated with rotamer change, including possible solvent effects. Incorporating these aspects goes beyond the scope of the present work and is indeed only addressed in the outlook of our paper.

To clarify this point in the manuscript, we extended the discussion in the Conclusions. That paragraph now reads:
"Moreover, in this study, we highlighted the dependence of the predictions on the side chain orientation by sampling alternative conformations for a single amino acid using the Richardson rotamer library [44]. This limitation is partly inherent to any approach based on single static structures. Possible improvements could involve extending the analysis to different rotamer libraries [54,55] and integrating the predictors with more extensive conformational sampling strategies, such as Monte Carlo methods or genetic algorithms optimization. Both approaches pose non-trivial challenges, as in Monte Carlo, suitable trial moves need to be established. Such moves must balance the increase in predictor probability with maintaining physically realistic conformations, avoiding steric clashes, and accounting for the energy cost of rotamer transitions, potentially including solvent effects."

8. The Ru-based probability map is derived from only 28 unique protein structures containing 47 Ru binding sites. The original Metal1D zinc map used approximately 2,085 structures with roughly 15,600 binding sites, a factor of ~75 more data than the original. I find it surprising that the authors present the Ru map as a usable predictive tool, given the sparse data. I would like the authors to provide a statistical analysis of the reliability of the Ru map. For example, a bootstrap or leave-one-out cross-validation demonstrates that the residue binding frequencies in Fig. 2 are stable and not an artefact of the small sample size. If the map is not statistically reliable, the authors should state this explicitly and caution against its use.
   We agree that the map containing only 47 binding sites is likely unreliable, but as explained in the first section of the results ("Differences in data availability"), this striking difference in the available number of structures "highlights how challenging the problem of designing a predictor for some TM binding sites can be due to the limited amount of data for training, especially in the case of 3DCNNs, and ML models in general." For this reason, we used this small dataset only for the Metal1D predictor, and even there, we noted in multiple cases that we obtained poorer results with that probability map. This is even clearer now that, thanks to the reviewer's suggestion, we added a table reporting TP/FN/recall for each site and predictor.

   The limitations are clearly stressed in the conclusions, where we mention "We observed some advantages in the case of the Pt-based probability map, but the Ru-based one was unsuccessful, possibly due to the extremely limited set of structures available for its generation". In the same section, we explicitly suggest that expanding the dataset by, e.g., combining related transition metals, would be a good strategy.

   To clarify this, we added an additional statement there, "and we discourage its use in the current form".

9. Throughout the manuscript, predicted probabilities from Metal3D are reported as single point values (e.g., p ~ 0.14, p ~ 0.04, p ~ 0.2), and Metal1D scores are reported to three decimal places (e.g., 0.032, 0.008). No uncertainty or variance is reported for any of these values. For Metal3D, the prediction is generated by averaging probability boxes across different Cαcentred windows, so in principle a variance can be computed. For Metal1D, the score depends on a geometric search that is sensitive to small coordinate perturbations. I think the authors should report either confidence intervals or a measure of score sensitivity to small coordinate perturbations (e.g., ±0.1 ˚A RMSD noise on the input structure).
We thank the reviewer for their suggestion. In principle, their suggested methods could be used to measure the “uncertainty” associated with the prediction. However, given the limited number of structures analyzed, we believe that a systematic uncertainty analysis would not substantially strengthen the conclusions drawn in this manuscript, and such an analysis would likely be more meaningful in the context of a larger-scale benchmark or statistical study across a broader dataset.
The aim of the present work is primarily exploratory and focuses on illustrating how predictive tools can be used for TM compounds and what the main challenges are. For these reasons, we have chosen to report the predicted scores as point estimates while keeping the discussion qualitative.

10. All conclusions about whether a site is “predicted” or “not predicted” by Metal3D rest on the choice of isovalue thresholds (0.04 for the lower contour; 0.14 or 0.16 for the higher contour). I would like the authors to perform a sensitivity analysis of the effects on precision and recall as the lower threshold is varied from 0.02 to 0.08. Is the qualitative conclusion that His15 is predicted but Asp101 and Asp119 are not robust across this range, or is it threshold-dependent? For Metal1D, an analogous analysis varying the relative threshold from 0.95 to 0.999 should be provided.
As explained in the method section, for Metal3D we set the isovalue threshold in such a way as to obtain distinct sites, but the low-probability regions remain our central focus here: increasing the isovalue would restrict the predictions to high-probability ones, but these are simply the ones more likely for Zn ions. In this study, the focus is different, as we want to consider all regions predicted and test if, in these low-probability regions, there is some prediction ability for TM-based drugs. Similarly, in the case of Metal1D, our choice of threshold is to include all predictions made. Setting a different relative threshold will only restrict the prediction to high-probability ones, which are not the only ones we focus on.

11. Metal1D scores each putative site by summing probabilities from its LINK-derived probability map over all compatible coordination environments within the search radius. I want to understand more clearly what physical quantity this score approximates. The LINK records in the PDB reflect which coordination environments happen to have been crystallised and deposited, and are therefore biased by crystallisation conditions, resolution limits, and the popularity of particular protein families as model systems. The score is not a binding free energy, a structural stability measure, or a thermodynamic quantity. Does this purely frequency-based score have predictive validity for identifying novel TM binding sites in proteins not represented in the training set? I think a more careful discussion of the statistical

nature and limitations of the Metal1D scoring function is needed, supported by a leave-one-protein-out validation.
As correctly noted, the score from Metal1D is not intended to approximate a thermodynamic quantity such as a binding free energy, but it is based on geometrical criteria with the main goal of having an accurate but fast predictor. The predictive validity of such a statistical approach was evaluated in the original Metal1D publication, where both predictors were thoroughly tested in different conditions and on large test sets, not included in the training data.

The underlying assumption behind the generation of probability maps from LINK records is that recurring coordination geometries and residue combinations reflect chemically favorable interactions. As the source of our data is the PDB data bank, like any other model based on such a database, we have an intrinsic bias toward more common structures and experimental conditions. One filtering step we do is to discard the same protein (retaining the one with the best score) in case it appears more than once, in order to avoid a further bias towards "popular proteins".

In the revised manuscript, we clarified the statistical nature of the Metal1D score by adding the following sentences in the methods section:
"These probability maps represent empirical coordination preferences derived from the frequency with which specific amino acids are observed to coordinate a given metal ion in experimentally determined structures in the PDB."

"In this way, higher scores correspond to environments that more closely resemble frequently observed coordination motifs for the metal considered.
The resulting scores indicate how consistent a local amino-acid environment is with known metal coordination patterns. In the limiting case where the local environment is compatible with all coordination motifs present in the probability map, the total probability sums up to 1. In practice, scores result between 0 and <1."

12. The manuscript benchmarks Metal3D and Metal1D against each other and against the known experimental binding sites, but never compares them against any established binding site detection tool such as fpocket, SiteMap (Schrödinger), or CASTp. I find this lack of baseline comparators a significant weakness: it is possible that a generic cavity-detection algorithm would achieve similar or better recall on the tested systems, since all tested binding sites involve surface-exposed or shallow clefts. I ask the authors to include at least one publicly available baseline (FPocket is free and widely used) and to report its precision and recall on the same test set under the same evaluation criteria.
We thank the reviewer for suggesting this comparison. We decided to include a new section in the manuscript ("Comparison with cavity-detection predictors") where we specifically test the cavity predictions of Fpocket for the HEWLC and RNaseA structures. This comparison highlighted that some of the sites can be identified by pocket predictors, but others are missed. We also computed site-specific TP/FP and recall, for comparison with the ones for Metal1D and Metal3D already added to the

SI:

Table S5: Summary of the true positive (TP), false negative (FN), and recall values for the sites identified with the cavity-detector predictor Fpocket. For each case, the values reported are: TP/FN (recall).

Protein	Site	Fpocket
HEWLC	His15	3/3 (0.50)
	Asp101	5/1 (0.83)
	Asp119	1/5 (0.17)
RNaseA	His119	1/3 (0.25)
	His105	0/4 (0.0)
	Asp14	0/4 (0.0)

We also extended the conclusion section to suggest that cavity-detection predictors could be combined with TM metal site predictors, as from our comparison, they seem to identify different regions.

All predictions performed with Fpocket, as well as VMD visualization states to generate Figure 8, have been added to a new version of the Zenodo repository.

13. Throughout the paper, a predicted site is considered a “hit” if a Metal3D probability region or a Metal1D coordinate is visually in proximity to the known binding amino acid. I find this criterion vague and inconsistently applied. What is the exact distance threshold used to call a prediction a true positive, that is, how close (in Å) must the predicted site centroid be to the known binding atom? Is this criterion identical for all three protein systems? I ask the authors to state this threshold explicitly and apply it uniformly.
We thank the reviewer for raising this point. Thanks to the additions in reply to question 4, and the additional table S1, this point should now be addressed. In the method section, we clearly report which distance threshold was used to classify a prediction as TP.

14. Predictions are primarily presented for apo structures, but the resolution of PDB 194L (the primary HEWLC apo reference) is not reported anywhere in the main text or in the SI RMSD tables. Since Metal3D uses atomic coordinates directly and the quality of side-chain orientations depends on resolution, I ask the authors to report the resolution of each PDB structure used in Tables S1 and S2 and to discuss whether any low-resolution structures may have introduced coordinate uncertainty that affects the predictions.
The values of the resolution have been added to the SI tables. All the structures consistently have good resolution (<1.90Å for the HEWLC case, and <2.27Å for RNaseA), and none of them have particularly low resolution that could have a strong influence on the predictions. All the values are below the resolution threshold used in the original Metal1D/3D publication of 2.5Å that was used to select structures from the PDB.

In the Methods section, we added this sentence to make this clear:
“In particular, Tables S2 and S3 report the resolution of all the PDB structures considered, showing that the structures in this dataset have uniformly resolutions

below the original threshold of 2.5 Å used to select crystal structures for the probability map generation of Metal1D and the training of Metal3D [12].”

Literature review and data reproducibility: I find the following citation issues significant enough to require correction before the manuscript can be considered complete. The data availability for this manuscript is better than average, as the authors have deposited predictions, visualisations, and a Jupyter notebook on Zenodo, which I commend. Nevertheless, I find several specific reproducibility gaps that must be addressed.

1. Reference [33] (Levy et al., bioRxiv 2025, doi:10.1101/2025.11.13.688318) is cited in the Methods as the primary source for the NCP GLU site test system. Since one of the authors of the current manuscript is also an author on Ref. [33], I ask the authors to confirm whether a peer-reviewed journal version is now available, and if so, to update the citation. Citing one’s own unpublished preprint as the primary source of experimental data for a test system raises concerns about reproducibility.
   The reference to our own work, currently under review, refers to the naming “GLU site” introduced to identify the Glu61 and Glu64 site in the H2A histone protein. The source of experimental data, as explained in the text, are deposited PDB structures (PDB IDs 3REH and 5XF3), which are available on the PDB database and correctly cited.

2. No comparison is made with established binding site predictors, and the relevant literature is not cited. If the authors choose not to include a numerical baseline comparison, they should at a minimum cite fpocket, SiteMap, and CASTp, and explicitly discuss why Metal3D and Metal1D are expected to outperform these tools for TM binding sites.
   In response to another comment from the reviewer, we have now included a comparison with the cavity detector Fpocket (see response to comment 12 and the new subsection “Comparison with cavity-detection predictors”). We also added the corresponding reference to the relevant literature. Concerning other detection tools, rather than citing only a few individual methods, we chose to refer to general publications discussing different methodologies used in the field, as we believe this provides a broader overview while avoiding an incomplete or selective list of specific predictors.

3. The manuscript cites the Richardson penultimate rotamer library [44] for rotamer sampling. Since backbone-dependent rotamers are a relevant methodological consideration, the Dunbrack & Cohen backbone-dependent rotamer library should also be cited and its non-use justified.
   We addressed this in reply to question 7, and this point should now be clear thanks to the additional modifications to the manuscript added to reply to the previous question of the reviewer.

4. No structure validation is reported for the PDB structures used as input. I ask the authors to cite MolProbity or an equivalent tool and to confirm that all structures used have acceptable geometry (Ramachandran outliers below 2%, rotamer outliers below 5%, clashscore below 20). This is particularly important for PDB 3REH and 5XF3, which are the sole basis for the bidentate GLU site analysis

Structural validation metrics were obtained from the wwPDB validation report available on the PDB website for each structure. All PDB structures used in this work are clearly cited in the manuscript, and we have now additionally reported their resolution in response to comment 14.
In all cases, the values are within acceptable ranges, including PDB 3REH and 5XF3 explicitly mentioned by the reviewer, used in the analysis of the bidentate GLU site.

Given that these metrics are readily available in the wwPDB validation reports and that all structures satisfy standard quality criteria, we believe including the full set of validation statistics in the manuscript or SI would not substantially improve clarity for the reader.
For completeness, in reply to the reviewer's comment, we report here the values that can be found on the PDB website from the wwPDB validation reports for the structures considered.

PDB ID	Resolution (Å)	Ramachandran outliers (%)	Sidechain outliers (%)	Clashscore
194L	1.40	0	3.8	6
2I6Z	1.90	0	4.8	5
5II3	1.78	0	1.0	4
5V4G	1.20	0	0	1
6QEA	1.96	0	1.0	5
6WGO	1.30	0	0	5
1RPH	2.20	1.6	7.4	21
4S0Q	2.09	0.4	2.4	5
4S18	2.27	0.4	2.4	4
5JLG	1.79	1.2	5.5	7
3REH	2.50	0.3	4.5	6
5XF3	2.60	0.7	4.6	8

5. Reference [43] (EVOLVE, ChemRxiv 2023) is cited for the rotamer sampling approach used in this work. I would like the authors to verify whether a peer-reviewed version of the EVOLVE paper is now available and, if so, to update the citation. Methodological choices should ideally be grounded in peer-reviewed sources.
The manuscript currently remains available only as a preprint. The EVOLVE package itself is openly available (http://lcbc.epfl.ch/evolve). In the present work, this reference is cited primarily to acknowledge the use of a similar rotamer sampling strategy based on the Richardson rotamer library. Since no peer-reviewed publication is available, we have kept the preprint citation.

6. The probability map generation from LINK records is described only briefly and deferred entirely to Ref. [12]. However, since the authors generate two new maps (Pt and Ru) in this paper using the same approach, and since the small dataset size raises questions about statistical validity, I think a brief self-contained description of the map generation procedure, including how LINK records were filtered, how duplicate structures were removed, and how probabilities were normalised and should be provided in the Methods or SI. This information is necessary for reproducibility.

   We thank the reviewer for pointing out this potential source of confusion for the reader. We added a more detailed description of the probability map generation to the manuscript in the methods section:

   "To construct a probability map, all relevant LINK records, including the metal of interest and protein atoms, are extracted. Next, the coordinating residues for each metal are converted into a unique coordination environment by associating one letter code and alphabetically sorting this code to group equivalent environments, e.g., CCH or CHC, etc. Duplicate PDB entries are removed to avoid over-representing frequently crystallized proteins, retaining the protein structure with the best resolution. The counts of each unique coordination environment across the filtered dataset are then normalized, producing a probability map that reflects the relative frequency of each observed coordination motif."

   This procedure is analogous to that of the original publication and was applied to generate the new probability maps for Pt and Ru in this work. To ensure reproducibility, we provide the probability maps generated, as well as a Jupyter notebook for the generation of probability maps from alternative PDB datasets.

7. The manuscript does not report version numbers for Metal3D, Metal1D, or VMD. Since both predictors are under active development, results may differ across versions. I ask the authors to report the exact versions of all software tools used, including the Metal3D/Metal1D package and the VMD versions, in the Methods section.
   We added the versions of Metal3D and Metal1D (v0.2) and VMD (v 1.9.3) in the manuscript.

8. The preprocessing steps applied to raw PDB structures before running the predictors are not described anywhere. Specifically, (a) were hydrogen atoms added, and if so, with which tool and at what pH, (b) were alternate conformations resolved, and if so, how, (c) were crystallographic water molecules and co-crystallised ligands removed from the input file or merely ignored by the code? The manuscript states that "non-protein atoms are not considered," but it is not clear whether they were physically removed from the input file. This distinction matters because some implementations may behave differently depending on whether extraneous atoms are present.
   Hydrogen atoms are not added, and both Metal3D and Metal1D use raw PDB structures and are solely based on the location of heavy atoms. Concerning

non-protein atoms, they are ignored by Metal3D and Metal1D, so there is no need to physically remove them from the input file. To clarify this, the sentence mentioned by the reviewer now reads "Moreover, even if the crystal structure includes non-protein atoms, such as ions, water molecules, or other ligands, they are not considered by Metal1D and Metal3D when performing a prediction, even if they are present in the input structure."

9. I ask the authors to confirm explicitly in the Methods which files are deposited on Zenodo, including (a) the probability map files for Pt and Ru in their native format, (b) the input PDB files as actually used after preprocessing, (c) the Metal3D global probability density files (.dx or .cube format) for all structures shown in the figures, and (d) the rotamer-sampled PDB coordinate files for the structures shown in Figs. 7 and 8. Without these files, an independent researcher cannot reproduce Figs. 4, 6, 7, and 8.
   We confirm that all the results are reported in the Zenodo repository, including probability map files and a Jupyter notebook for their generation, also in case of a new dataset (as already stated in the method section).
   The same applies to the conformers, for which another Jupyter notebook is provided for their generation, and all the structures and predictions are available. In the same repository, the PDB used for predictions as well as Metal3D global probabilities (cube files) and Metal1D located sites are present. For the detailed list of files, we consider the "data availability statement" section more appropriate.

10. The rotamer sampling procedure is described as generating conformations from the Richardson library, but no random seed is reported. If the sampling involves any stochastic element (e.g., random selection among rotamers of similar probability), results would not be exactly reproducible without a fixed seed. I ask the authors to clarify whether the sampling is entirely deterministic or stochastic, and if stochastic, to report the random seed used.
    The rotamer sampling involved testing all the rotamers present in the library, replacing the amino acid under study. This does not involve any stochastic aspect.

11. The exact Metal1D search radius used in this study is not reported. The original Ref. [12] describes a default value, but since the organic ligands coordinating the TM center impose additional steric constraints, I want to confirm whether the default search radius was used or adjusted for TM complexes. This parameter must be stated explicitly in the Methods.
    We thank the reviewer for raising this concern. Indeed, the default value of 5.5Å was used, as it is related to the distance between the metal ion and the coordinating atom in the protein, which remains the typical TM-atom distance even in the presence of organic ligands.
    For clarity, we added the following sentence to the Methods section:
    "Another parameter specific to Metal1D is the search radius, defined as twice the distance between the metal ion and the coordinating atom of the amino acid. By default, Metal1D uses a search radius of Å. The same value was used for all Metal1D predictions in this study, as it exceeds the ideal coordination distance, thereby accounting for deviations from ideal coordination geometries."
